# Peculiarity of Pharmaceutical Marketing in Serbia

**DOI:** 10.3389/fpubh.2015.00150

**Published:** 2015-06-25

**Authors:** Veselin Dickov

**Affiliations:** ^1^Institute for the Health Protection of Students, Novi Sad, Serbia

**Keywords:** pharmaceutical market, pharmaceutical industry, pricing regulations, strategic analysis

Major challenge for the Western Balkan pharmaceutical markets remains legal enforcement of measurable tender criteria (1) when submitting offers for supplying large healthcare centers. All of regional economies increased health spending rapidly over past two and a half decades. Nevertheless, constrained national drug budgets have led to the development of diverse resource allocation strategies (2). Public debate has created an impression that there is room for more active inclusion of marketing in generating strategic decisions at the company level (3). Focus on operative, daily duties may also be the result of the complex and difficult conditions in which the pharmaceutical industry functions in Serbia (4). This market is characterized by modest size, decent growth rates, and heavy domination of generics as compared to other Eastern European countries (5). Local industry balances between imposed legislative limitations, its own orientation to generating profit, and *de facto* present social and ethical factor. Value creation in domestic market is unified in synergetic effect of the product, pricing, distribution, and promotion (6). A superior competitive strategy must be sought at the enhanced product level, i.e., in the concept of value added, where companies on the market of Serbia are only making their first steps (7).

A networked environment may serve as a useful framework for a brand new view of the roles of individual stakeholders, once again with the aim to maximize the outputs of healthcare system. There is also the traditional lag existing in the arrival of innovation to the market of Serbia. It is not characteristic of the pharmaceutical industry, but rather results from the fact that recent years have seen an evident lag of Serbian economy behind global trends, manifested in the delayed effect of the global economic crisis (8). Suppliers on the Serbian market argue that patient education is the responsibility of the healthcare system. The practices of pharmaceutical companies in the markets of developed countries testify to a new, more active role of the pharmaceutical industry in this education (9). Opinions on direct promotion speak that originators of supply on the Serbian market do not see patients as “legitimate target” of the industry’s marketing effort, which is in accordance with the marketing practices elsewhere in liberal, less regulated, high-income markets (10).

Based on published evidence, it can be concluded that there is a significant potential for improving the marketing function, its place and role, and the resulting marketing activities (11). As cited, contemporary research included 30 companies on the territory of the Europe, the problems faced, and conclusions presented correspond closely to the results of qualitative research conducted on 10 participants on the pharmaceutical market of Serbia (12).The common premise is that the European market is far more developed and matured in terms of the application of marketing. Government regulations on pricing and the objective permanence of distribution channels prevent pharmaceutical companies from viewing them as active marketing mix elements. The socio-political element is especially manifested in the domain of price control (13). Formalized process of adopting marketing strategies may be potentially successful in certain market conditions and specific corporate culture. Change in conditions may valorize different approaches to planning and implementing marketing strategies. It appears that different degrees of formalization of adopting marketing strategies and a different position and role of marketing within an organization may result in equally successful pharmaceutical companies. Companies that participated in the research in Serbia achieved their leading position through successful adaptation to changed conditions. Nevertheless, the process of transformation in the environment and the organization is permanent (14). Some of the respondents very objectively assessed that changes in the business conditions and organization also imply new behavior patterns in order to accomplish the objectives (15). Further acceleration of population aging and growth of global emerging markets reshaping health care landscape worldwide will give the long-term imprint to the pharmaceutical markets evolution in small nations bordering EU’s economic zone (16).

## Conflict of Interest Statement

The author declares that the research was conducted in the absence of any commercial or financial relationships that could be construed as a potential conflict of interest.
